# 6,6′-Dibromo-2,2′-dihex­yloxy-1,1′-bi­naphthalene

**DOI:** 10.1107/S1600536808033369

**Published:** 2008-10-18

**Authors:** Yu-Feng Li, Lin-Tong Wang, Fang-Fang Jian

**Affiliations:** a Microscale Science Institute Weifang University, Weifang 261061, People’s Republic of China; b Department of Chemistry and Chemical Engineering, Weifang University, Weifang 261061, People’s Republic of China

## Abstract

The title compound, C_32_H_36_Br_2_O_2_, was prepared by the reaction of 6-bromo-1-(2-bromo-6-hydroxy­naphthalen-5-yl)­naphthalen-2-ol and 1-iodo­hexane. The dihedral angle between the naphthalene ring planes is 63.8 (9)° The crystal structure may be stabilized by two very weak π–π inter­actions involving the six-membered rings, with centroid–centroid distances of 4.012 (4) and 4.010 (4) Å. The crystal studied was an inversion twin.

## Related literature

For applications of 6,6′-dibromo-1,1′-bi-2-naphthol derivatives, see: Hu *et al.* (1996 ▶). For bond-length data, see: Vannes & Vos (1978 ▶).
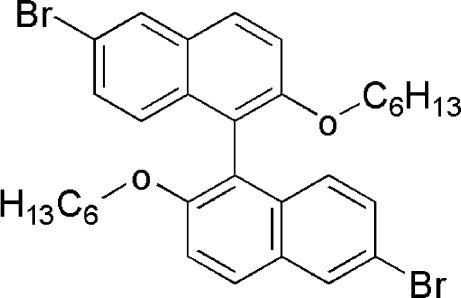


## Experimental

### 

#### Crystal data

C_32_H_36_Br_2_O_2_
*M* *_r_* = 612.43
Orthorhombic, 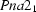

*a* = 12.401 (3) Å
*b* = 8.1742 (16) Å
*c* = 27.396 (6) Å
*V* = 2777.1 (10) Å^3^
*Z* = 4
Mo *K*α radiation
μ = 2.95 mm^−1^
*T* = 293 (2) K
0.25 × 0.20 × 0.18 mm

#### Data collection

Bruker SMART CCD area-detector diffractometer
Absorption correction: none
26425 measured reflections
3647 independent reflections
3581 reflections with *I* > 2σ(*I*)
*R*_int_ = 0.062

#### Refinement

*R*[*F* ^2^ > 2σ(*F* ^2^)] = 0.036
*wR*(*F* ^2^) = 0.103
*S* = 0.91
3647 reflections
325 parameters
1 restraint
H-atom parameters constrained
Δρ_max_ = 0.53 e Å^−3^
Δρ_min_ = −0.38 e Å^−3^
Absolute structure: Flack (1983 ▶), with 2766 Freidel pairs
Flack parameter: 0.49 (2)

### 

Data collection: *SMART* (Bruker, 1997 ▶); cell refinement: *SAINT* (Bruker, 1997 ▶); data reduction: *SAINT*; program(s) used to solve structure: *SHELXS97* (Sheldrick, 2008 ▶); program(s) used to refine structure: *SHELXL97* (Sheldrick, 2008 ▶); molecular graphics: *SHELXTL* (Sheldrick, 2008 ▶); software used to prepare material for publication: *SHELXTL*.

## Supplementary Material

Crystal structure: contains datablocks global, I. DOI: 10.1107/S1600536808033369/at2650sup1.cif

Structure factors: contains datablocks I. DOI: 10.1107/S1600536808033369/at2650Isup2.hkl

Additional supplementary materials:  crystallographic information; 3D view; checkCIF report

## Figures and Tables

**Fig. 1.**
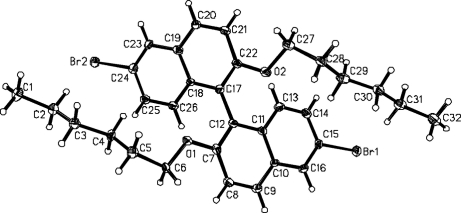
The structure of the title compound showing 30% probability displacement ellipsoids and the atom-numbering scheme.

## supplementary crystallographic information

### Comment

6,6'-Dibromo-1,1'-bi-2-naphthol derivatives have received considerable attention in the literature. They are attractive from several points of view in application (Hu *et al.*, 1996). As part of our search for new 6,6'-dibromo-1,1'-bi-2-naphthol compounds we synthesized the title compound (I), and describe its structure here. The angle between the planes of the naphthalene rings is 60.1 (6)°.

The C17—C12 bond length of 1.504 (6)Å is comparable with C—C single bond [1.532 (4) Å] reported (van Nes *et al.*, 1978). In the structure, there is no classcical hydrogen bonds. The crystal structure is stabilized by weak π-π interactions involving the six-membered rings (Cg1: C7-C12) and (Cg4^î^: C18-C26) [ (i) 1/2 + x, 3/2 - y, z], and the other six-membered six-membered rings (Cg2: C10-C16) and (Cg3^î^: C17-C22) rings, with centroid-centroid distances of 4.012 (4) \%A and 4.010 (4) Å, respectively.

### Experimental

A mixture of the 6-bromo-1-(2-bromo-6-hydroxynaphthalen-5-yl) naphthalen-2-ol (0.1 mol), and 1-iodohexane (0.22 mol) was stirred in refluxing acetone/K_2_CO_3_ mixture (20 mL) for 4 h to afford the title compound (0.086 mol, yield 86%). Single crystals suitable for X-ray measurements were obtained by recrystallization from ethanol at room temperature.

### Refinement

H atoms were fixed geometrically and allowed to ride on their attached atoms, with C—H distances = 0.93 - 0.97 Å, and with *U*_iso_=1.2–1.5*U*_eq_(C,N).

### Crystal data

**Table:**

C_32_H_36_Br_2_O_2_	*F*(000) = 1256
*M**_r_* = 612.43	*D*_x_ = 1.465 Mg m^−^^3^
Orthorhombic, *P**n**a*2_1_	Mo *K*α radiation, λ = 0.71073 Å
Hall symbol: P 2c -2n	Cell parameters from 1536 reflections
*a* = 12.401 (3) Å	θ = 2.5–23.6°
*b* = 8.1742 (16) Å	µ = 2.95 mm^−^^1^
*c* = 27.396 (6) Å	*T* = 293 K
*V* = 2777.1 (10) Å^3^	Block, yellow
*Z* = 4	0.25 × 0.20 × 0.18 mm

### Data collection

**Table:**

Bruker SMART CCD area-detector diffractometer	3581 reflections with *I* > 2σ(*I*)
Radiation source: fine-focus sealed tube	*R*_int_ = 0.062
graphite	θ_max_ = 27.5°, θ_min_ = 1.5°
φ and ω scans	*h* = −16→16
26425 measured reflections	*k* = −10→10
3647 independent reflections	*l* = −35→35

### Refinement

**Table:**

Refinement on *F*^2^	Secondary atom site location: difference Fourier map
Least-squares matrix: full	Hydrogen site location: inferred from neighbouring sites
*R*[*F*^2^ > 2σ(*F*^2^)] = 0.036	H-atom parameters constrained
*wR*(*F*^2^) = 0.103	*w* = 1/[σ^2^(*F*_o_^2^) + (0.031*P*)^2^] where *P* = (*F*_o_^2^ + 2*F*_c_^2^)/3
*S* = 0.91	(Δ/σ)_max_ = 0.002
3647 reflections	Δρ_max_ = 0.53 e Å^−^^3^
325 parameters	Δρ_min_ = −0.38 e Å^−^^3^
1 restraint	Absolute structure: Flack (1983), 2766 Freidel pairs
Primary atom site location: structure-invariant direct methods	Flack parameter: 0.49 (2)

### Special details

**Table:**

Geometry. All e.s.d.'s (except the e.s.d. in the dihedral angle between two l.s. planes) are estimated using the full covariance matrix. The cell e.s.d.'s are taken into account individually in the estimation of e.s.d.'s in distances, angles and torsion angles; correlations between e.s.d.'s in cell parameters are only used when they are defined by crystal symmetry. An approximate (isotropic) treatment of cell e.s.d.'s is used for estimating e.s.d.'s involving l.s. planes.
Refinement. Refinement of *F*^2^ against ALL reflections. The weighted *R*-factor *wR* and goodness of fit *S* are based on *F*^2^, conventional *R*-factors *R* are based on *F*, with *F* set to zero for negative *F*^2^. The threshold expression of *F*^2^ > σ(*F*^2^) is used only for calculating *R*-factors(gt) *etc*. and is not relevant to the choice of reflections for refinement. *R*-factors based on *F*^2^ are statistically about twice as large as those based on *F*, and *R*- factors based on ALL data will be even larger.

### Fractional atomic coordinates and isotropic or equivalent isotropic displacement parameters (Å^2^)

**Table:**

	*x*	*y*	*z*	*U*_iso_*/*U*_eq_	
Br1	0.72348 (8)	0.71557 (12)	0.21977 (2)	0.0350 (2)	
Br2	0.02939 (8)	0.71401 (13)	0.52428 (2)	0.0354 (2)	
C25	0.2283 (6)	0.7910 (11)	0.4792 (4)	0.0289 (19)	
H25A	0.2582	0.7353	0.5055	0.035*	
O1	0.4163 (4)	1.1960 (8)	0.4440 (2)	0.0311 (12)	
O2	0.3351 (4)	1.1966 (8)	0.2989 (2)	0.0314 (12)	
C30	0.5435 (7)	1.2962 (13)	0.1715 (4)	0.0310 (19)	
H30A	0.5845	1.3763	0.1899	0.037*	
H30B	0.5045	1.3541	0.1461	0.037*	
C14	0.5233 (6)	0.7941 (12)	0.2653 (4)	0.0297 (19)	
H14A	0.4927	0.7390	0.2390	0.036*	
C3	0.2086 (7)	1.2922 (12)	0.5727 (4)	0.031 (2)	
H3A	0.2469	1.3490	0.5986	0.037*	
H3B	0.1663	1.3727	0.5552	0.037*	
C6	0.4604 (9)	1.2618 (8)	0.4870 (4)	0.019 (2)	
H6A	0.5139	1.3441	0.4789	0.022*	
H6B	0.4956	1.1761	0.5056	0.022*	
C24	0.1149 (5)	0.8051 (10)	0.4732 (3)	0.0267 (16)	
C27	0.2915 (10)	1.2656 (11)	0.2526 (5)	0.040 (3)	
H27A	0.2371	1.3475	0.2595	0.048*	
H27B	0.2591	1.1798	0.2330	0.048*	
C8	0.5971 (6)	1.1092 (9)	0.4195 (3)	0.0295 (17)	
H8A	0.6277	1.1601	0.4465	0.035*	
C16	0.6830 (5)	0.8758 (9)	0.3093 (3)	0.0272 (16)	
H16A	0.7577	0.8774	0.3124	0.033*	
C10	0.6174 (5)	0.9496 (9)	0.3456 (3)	0.0223 (16)	
C15	0.6363 (5)	0.8027 (11)	0.2699 (3)	0.0253 (15)	
C11	0.5035 (5)	0.9499 (8)	0.3400 (3)	0.0226 (15)	
C29	0.4653 (8)	1.2176 (11)	0.2043 (4)	0.038 (2)	
H29A	0.5039	1.1641	0.2306	0.046*	
H29B	0.4262	1.1342	0.1864	0.046*	
C4	0.2923 (7)	1.2171 (10)	0.5372 (3)	0.0260 (19)	
H4A	0.3319	1.1320	0.5542	0.031*	
H4B	0.2543	1.1662	0.5102	0.031*	
C19	0.1345 (6)	0.9496 (9)	0.3975 (3)	0.0257 (17)	
C22	0.2678 (6)	1.1123 (8)	0.3292 (2)	0.0258 (16)	
C17	0.3157 (5)	1.0338 (9)	0.3686 (3)	0.0260 (17)	
C23	0.0695 (5)	0.8747 (9)	0.4339 (3)	0.0275 (16)	
H23A	−0.0051	0.8739	0.4305	0.033*	
C32	0.7062 (12)	1.2663 (12)	0.1191 (6)	0.051 (4)	
H32A	0.7554	1.1890	0.1050	0.076*	
H32B	0.7450	1.3386	0.1405	0.076*	
H32C	0.6725	1.3288	0.0937	0.076*	
C12	0.4362 (5)	1.0324 (9)	0.3741 (2)	0.0236 (17)	
C28	0.3838 (5)	1.3404 (8)	0.2262 (3)	0.0317 (17)	
H28A	0.4220	1.4119	0.2485	0.038*	
H28B	0.3556	1.4078	0.2000	0.038*	
C9	0.6621 (6)	1.0310 (10)	0.3865 (3)	0.0292 (18)	
H9A	0.7364	1.0314	0.3911	0.035*	
C18	0.2499 (5)	0.9489 (9)	0.4030 (3)	0.0228 (15)	
C20	0.0909 (6)	1.0301 (9)	0.3560 (3)	0.0270 (18)	
H20A	0.0166	1.0290	0.3513	0.032*	
C7	0.4848 (6)	1.1134 (8)	0.4131 (2)	0.0248 (16)	
C21	0.1543 (6)	1.1093 (9)	0.3226 (3)	0.0295 (17)	
H21A	0.1232	1.1607	0.2958	0.035*	
C5	0.3706 (5)	1.3383 (8)	0.5173 (3)	0.0321 (17)	
H5A	0.4026	1.3981	0.5442	0.038*	
H5B	0.3319	1.4163	0.4972	0.038*	
C13	0.4596 (6)	0.8659 (8)	0.2989 (2)	0.0284 (16)	
H13A	0.3851	0.8605	0.2952	0.034*	
C26	0.2926 (6)	0.8645 (8)	0.4437 (2)	0.0273 (16)	
H26A	0.3671	0.8578	0.4470	0.033*	
C31	0.6215 (6)	1.1766 (9)	0.1477 (3)	0.0332 (18)	
H31A	0.6557	1.1106	0.1727	0.040*	
H31B	0.5821	1.1040	0.1261	0.040*	
C2	0.1328 (6)	1.1696 (10)	0.5956 (3)	0.0362 (19)	
H2A	0.1740	1.0951	0.6159	0.043*	
H2B	0.0988	1.1059	0.5701	0.043*	
C1	0.0439 (10)	1.2518 (8)	0.6272 (6)	0.033 (3)	
H1A	−0.0008	1.1690	0.6417	0.049*	
H1B	0.0005	1.3216	0.6070	0.049*	
H1C	0.0770	1.3157	0.6525	0.049*	

### Atomic displacement parameters (Å^2^)

**Table:**

	*U*^11^	*U*^22^	*U*^33^	*U*^12^	*U*^13^	*U*^23^
Br1	0.0306 (5)	0.0454 (4)	0.0291 (5)	0.0060 (5)	0.0042 (4)	0.0017 (9)
Br2	0.0305 (5)	0.0448 (4)	0.0308 (5)	−0.0055 (5)	0.0024 (4)	0.0072 (9)
C25	0.027 (4)	0.029 (4)	0.031 (5)	0.000 (4)	−0.004 (3)	−0.006 (5)
O1	0.026 (3)	0.046 (3)	0.022 (3)	0.003 (3)	−0.003 (2)	−0.007 (3)
O2	0.027 (3)	0.041 (3)	0.026 (3)	−0.002 (3)	−0.004 (2)	0.010 (3)
C30	0.033 (5)	0.034 (4)	0.026 (5)	0.000 (5)	−0.005 (3)	−0.005 (5)
C14	0.033 (5)	0.034 (4)	0.022 (5)	0.000 (4)	−0.003 (3)	−0.007 (5)
C3	0.031 (5)	0.032 (4)	0.030 (5)	0.006 (4)	0.001 (3)	−0.010 (5)
C6	0.024 (5)	0.024 (5)	0.008 (4)	−0.003 (3)	0.002 (3)	0.000 (3)
C24	0.028 (4)	0.023 (4)	0.029 (4)	−0.004 (4)	0.004 (3)	−0.006 (4)
C27	0.041 (7)	0.042 (6)	0.037 (7)	0.009 (4)	−0.009 (5)	0.013 (4)
C8	0.029 (4)	0.037 (5)	0.022 (4)	−0.004 (3)	−0.003 (3)	0.000 (3)
C16	0.018 (3)	0.033 (4)	0.031 (4)	0.000 (3)	−0.002 (3)	0.007 (3)
C10	0.016 (4)	0.029 (4)	0.021 (4)	−0.001 (3)	−0.001 (3)	0.009 (3)
C15	0.027 (4)	0.029 (4)	0.020 (4)	0.004 (4)	0.006 (3)	−0.002 (4)
C11	0.023 (4)	0.022 (4)	0.022 (4)	−0.001 (3)	−0.004 (3)	0.003 (3)
C29	0.043 (5)	0.026 (4)	0.046 (6)	0.010 (5)	−0.017 (4)	−0.005 (5)
C4	0.025 (4)	0.032 (4)	0.021 (4)	0.008 (4)	0.010 (3)	−0.010 (4)
C19	0.027 (4)	0.024 (4)	0.026 (4)	0.002 (3)	−0.005 (3)	−0.006 (3)
C22	0.026 (4)	0.028 (4)	0.023 (4)	0.008 (3)	−0.007 (4)	−0.003 (3)
C17	0.022 (4)	0.032 (4)	0.024 (4)	0.001 (3)	0.001 (3)	−0.005 (3)
C23	0.020 (3)	0.035 (4)	0.028 (4)	−0.003 (3)	0.005 (3)	−0.008 (3)
C32	0.050 (9)	0.073 (10)	0.030 (7)	0.007 (5)	−0.008 (6)	−0.001 (5)
C12	0.019 (4)	0.032 (4)	0.020 (4)	−0.001 (3)	−0.005 (3)	0.004 (3)
C28	0.036 (4)	0.036 (4)	0.022 (4)	−0.002 (3)	−0.008 (4)	0.007 (3)
C9	0.021 (4)	0.039 (5)	0.028 (5)	0.000 (3)	−0.004 (3)	0.004 (4)
C18	0.017 (3)	0.031 (4)	0.020 (4)	0.005 (3)	−0.001 (3)	−0.006 (3)
C20	0.022 (4)	0.034 (5)	0.026 (5)	0.004 (3)	−0.003 (3)	−0.004 (4)
C7	0.031 (4)	0.023 (4)	0.020 (3)	0.004 (3)	0.001 (3)	0.001 (3)
C21	0.025 (4)	0.036 (5)	0.027 (4)	0.002 (3)	−0.007 (3)	−0.003 (3)
C5	0.032 (4)	0.031 (4)	0.033 (4)	0.002 (3)	0.004 (4)	−0.004 (4)
C13	0.020 (3)	0.033 (4)	0.032 (4)	−0.002 (3)	−0.003 (3)	0.006 (3)
C26	0.024 (4)	0.031 (4)	0.027 (4)	0.001 (3)	−0.005 (3)	−0.004 (3)
C31	0.037 (4)	0.028 (5)	0.035 (5)	−0.003 (3)	−0.007 (4)	0.001 (3)
C2	0.033 (4)	0.045 (6)	0.030 (5)	0.003 (3)	0.003 (3)	−0.003 (4)
C1	0.035 (7)	0.021 (7)	0.043 (8)	−0.001 (3)	0.011 (6)	−0.005 (3)

### Geometric parameters (Å, °)

**Table:**

Br1—C15	1.887 (7)	C29—H29A	0.9700
Br2—C24	1.907 (8)	C29—H29B	0.9700
C25—C26	1.393 (12)	C4—C5	1.491 (11)
C25—C24	1.421 (10)	C4—H4A	0.9700
C25—H25A	0.9300	C4—H4B	0.9700
O1—C7	1.377 (8)	C19—C20	1.420 (9)
O1—C6	1.406 (12)	C19—C23	1.422 (9)
O2—C22	1.363 (8)	C19—C18	1.438 (9)
O2—C27	1.491 (14)	C22—C17	1.388 (9)
C30—C29	1.470 (14)	C22—C21	1.420 (11)
C30—C31	1.522 (12)	C17—C18	1.427 (9)
C30—H30A	0.9700	C17—C12	1.503 (6)
C30—H30B	0.9700	C23—H23A	0.9300
C14—C13	1.349 (11)	C32—C31	1.501 (15)
C14—C15	1.408 (10)	C32—H32A	0.9600
C14—H14A	0.9300	C32—H32B	0.9600
C3—C2	1.510 (11)	C32—H32C	0.9600
C3—C4	1.550 (12)	C12—C7	1.393 (9)
C3—H3A	0.9700	C28—H28A	0.9700
C3—H3B	0.9700	C28—H28B	0.9700
C6—C5	1.524 (12)	C9—H9A	0.9300
C6—H6A	0.9700	C18—C26	1.415 (9)
C6—H6B	0.9700	C20—C21	1.368 (10)
C24—C23	1.342 (10)	C20—H20A	0.9300
C27—C28	1.485 (14)	C21—H21A	0.9300
C27—H27A	0.9700	C5—H5A	0.9700
C27—H27B	0.9700	C5—H5B	0.9700
C8—C9	1.368 (10)	C13—H13A	0.9300
C8—C7	1.404 (11)	C26—H26A	0.9300
C8—H8A	0.9300	C31—H31A	0.9700
C16—C15	1.364 (10)	C31—H31B	0.9700
C16—C10	1.419 (10)	C2—C1	1.554 (14)
C16—H16A	0.9300	C2—H2A	0.9700
C10—C9	1.417 (9)	C2—H2B	0.9700
C10—C11	1.420 (9)	C1—H1A	0.9600
C11—C12	1.423 (9)	C1—H1B	0.9600
C11—C13	1.427 (9)	C1—H1C	0.9600
C29—C28	1.546 (11)		
			
C26—C25—C24	116.8 (8)	C17—C22—C21	121.0 (6)
C26—C25—H25A	121.6	C22—C17—C18	119.6 (6)
C24—C25—H25A	121.6	C22—C17—C12	120.5 (6)
C7—O1—C6	117.6 (6)	C18—C17—C12	119.9 (6)
C22—O2—C27	119.2 (7)	C24—C23—C19	120.5 (7)
C29—C30—C31	113.6 (8)	C24—C23—H23A	119.7
C29—C30—H30A	108.8	C19—C23—H23A	119.7
C31—C30—H30A	108.8	C31—C32—H32A	109.5
C29—C30—H30B	108.8	C31—C32—H32B	109.5
C31—C30—H30B	108.8	H32A—C32—H32B	109.5
H30A—C30—H30B	107.7	C31—C32—H32C	109.5
C13—C14—C15	120.0 (8)	H32A—C32—H32C	109.5
C13—C14—H14A	120.0	H32B—C32—H32C	109.5
C15—C14—H14A	120.0	C7—C12—C11	118.3 (6)
C2—C3—C4	114.5 (8)	C7—C12—C17	120.3 (6)
C2—C3—H3A	108.6	C11—C12—C17	121.4 (6)
C4—C3—H3A	108.6	C27—C28—C29	115.2 (7)
C2—C3—H3B	108.6	C27—C28—H28A	108.5
C4—C3—H3B	108.6	C29—C28—H28A	108.5
H3A—C3—H3B	107.6	C27—C28—H28B	108.5
O1—C6—C5	109.2 (8)	C29—C28—H28B	108.5
O1—C6—H6A	109.8	H28A—C28—H28B	107.5
C5—C6—H6A	109.8	C8—C9—C10	120.8 (7)
O1—C6—H6B	109.8	C8—C9—H9A	119.6
C5—C6—H6B	109.8	C10—C9—H9A	119.6
H6A—C6—H6B	108.3	C26—C18—C17	123.0 (6)
C23—C24—C25	122.8 (8)	C26—C18—C19	117.2 (7)
C23—C24—Br2	121.4 (5)	C17—C18—C19	119.8 (6)
C25—C24—Br2	115.8 (6)	C21—C20—C19	122.3 (7)
O2—C27—C28	107.0 (9)	C21—C20—H20A	118.8
O2—C27—H27A	110.3	C19—C20—H20A	118.8
C28—C27—H27A	110.3	O1—C7—C12	115.9 (6)
O2—C27—H27B	110.3	O1—C7—C8	123.1 (6)
C28—C27—H27B	110.3	C12—C7—C8	120.9 (6)
H27A—C27—H27B	108.6	C20—C21—C22	119.5 (7)
C9—C8—C7	120.8 (7)	C20—C21—H21A	120.2
C9—C8—H8A	119.6	C22—C21—H21A	120.2
C7—C8—H8A	119.6	C4—C5—C6	113.7 (6)
C15—C16—C10	119.8 (6)	C4—C5—H5A	108.8
C15—C16—H16A	120.1	C6—C5—H5A	108.8
C10—C16—H16A	120.1	C4—C5—H5B	108.8
C9—C10—C16	122.0 (6)	C6—C5—H5B	108.8
C9—C10—C11	118.2 (7)	H5A—C5—H5B	107.7
C16—C10—C11	119.7 (6)	C14—C13—C11	121.7 (7)
C16—C15—C14	121.0 (7)	C14—C13—H13A	119.2
C16—C15—Br1	119.9 (5)	C11—C13—H13A	119.2
C14—C15—Br1	119.1 (6)	C25—C26—C18	123.1 (7)
C10—C11—C12	120.9 (6)	C25—C26—H26A	118.5
C10—C11—C13	117.6 (7)	C18—C26—H26A	118.5
C12—C11—C13	121.5 (6)	C32—C31—C30	110.8 (8)
C30—C29—C28	112.6 (8)	C32—C31—H31A	109.5
C30—C29—H29A	109.1	C30—C31—H31A	109.5
C28—C29—H29A	109.1	C32—C31—H31B	109.5
C30—C29—H29B	109.1	C30—C31—H31B	109.5
C28—C29—H29B	109.1	H31A—C31—H31B	108.1
H29A—C29—H29B	107.8	C3—C2—C1	112.7 (7)
C5—C4—C3	113.8 (7)	C3—C2—H2A	109.0
C5—C4—H4A	108.8	C1—C2—H2A	109.0
C3—C4—H4A	108.8	C3—C2—H2B	109.0
C5—C4—H4B	108.8	C1—C2—H2B	109.0
C3—C4—H4B	108.8	H2A—C2—H2B	107.8
H4A—C4—H4B	107.7	C2—C1—H1A	109.5
C20—C19—C23	123.0 (6)	C2—C1—H1B	109.5
C20—C19—C18	117.7 (7)	H1A—C1—H1B	109.5
C23—C19—C18	119.2 (7)	C2—C1—H1C	109.5
O2—C22—C17	116.4 (6)	H1A—C1—H1C	109.5
O2—C22—C21	122.6 (6)	H1B—C1—H1C	109.5
